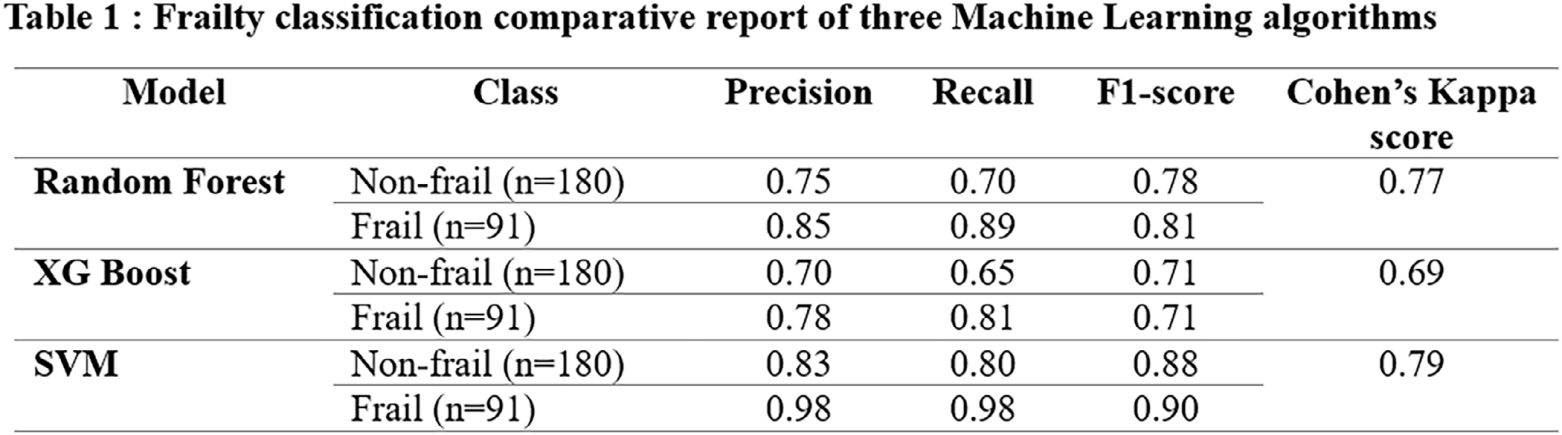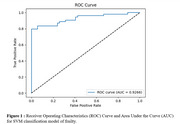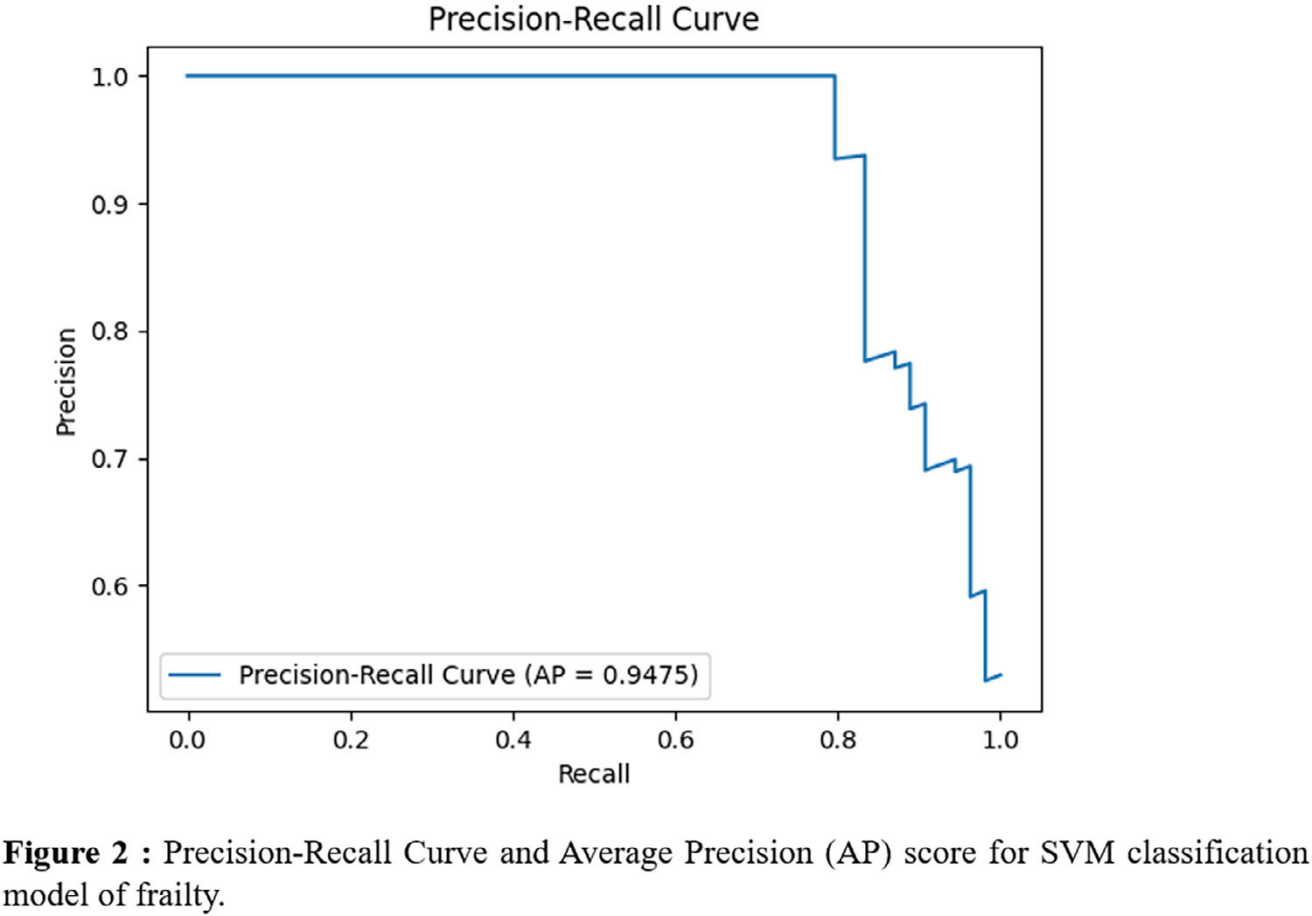# Frailty detection in older adults using speech‐based machine learning models : Potential of an accessible tool to limit neurocognitive disorders

**DOI:** 10.1002/alz70856_104419

**Published:** 2025-12-25

**Authors:** Eloïse DA Cunha, Raphael Zory, Frédéric Chorin, Valeria Manera, Auriane Gros

**Affiliations:** ^1^ Interdisciplinary Institute of Artificial Intelligence, Université Côte d'Azur, Nice, Alpes Maritimes, France; ^2^ CoBTeK Laboratory (Cognition Behaviour Technology), Nice, Alpes Maritimes, France; ^3^ Speech and Language Pathology Department, Université Côte d'Azur, Nice, Alpes Maritimes, France; ^4^ University Hospital Center of Nice, Nice, Alpes Maritimes, France; ^5^ Université Côte d'Azur, LAMHESS laboratory (Laboratoire Motricité Humaine Expertise Sport Santé), Nice, Alpes Maritimes, France; ^6^ Université Cote d'Azur, LAMHESS laboratory (Laboratoire Motricité Humaine Expertise Sport Santé), Nice, Alpes Maritimes, France

## Abstract

**Background:**

Geriatric physical frailty is a reversible condition characterized by a decline in physical and motor functions, significantly increasing the risk of neurocognitive disorders such as Alzheimer's disease (AD). During aging, associations between reduced physical capacities and neurocognitive impairment highlight the need for early detection to enable preventive interventions. Speech markers have shown potential in identifying neurodegenerative pathologies, yet their role in frailty detection remains unexplored. This study evaluates the effectiveness of machine learning models using speech‐based features for scalable, non‐invasive frailty screening in aging populations.

**Method:**

271 participants of 65 years and older were recruited from a university hospital (mean age 74.87±9.73; 63% female). Participants underwent a physical evaluation (endurance, strength, power, balance, body composition) and cognitive/behavioral assessments (MMSE, fatigue, lifestyle questionnaires). Frailty was scored using the Fried Frailty Index, considering five indices: unintentional weight loss, grip strength, exhaustion, low physical activity, and slow walking speed. Participants meeting three or more criteria were classified as frail (*n* = 91). Each participant recorded one‐minute spontaneous speeches about positive and then negative memories, from which acoustic, temporal, and linguistic features were automatically extracted. Three machine learning models (Random Forest, XGBoost, and Support Vector Machine (SVM)) were trained and tested using a 70/30 data‐split to classify the frailty status.

**Results:**

The SVM model demonstrates the highest classification capacity outperforming other tested models. The ROC curve illustrates the model's strong balance between sensitivity and specificity (AUC=0.93), while the Precision‐Recall curve (AP=0.95) underscores its robustness in maintaining high precision across recall levels. Therefore, SVM demonstrates robust interest for speech‐based frailty detection, highlighting speech analysis as an effective marker in the elderly population, using both acoustic, temporal and linguistic markers.

**Conclusions:**

Speech‐based machine learning models appear as the first accessible and scalable method for early frailty screening. This scalable approach can facilitate timely interventions to restore robustness, ultimately reducing the risk of neurocognitive disorders. Implementing speech‐based frailty classifiers in clinical settings may enhance preventive strategies for aging populations, optimizing care pathways and reducing neurocognitive risks. Further studies with larger, more diverse cohorts and advanced methodologies are essential to validate and generalize these promising findings in real‐world contexts.